# Unilateral Pushup-Induced Acute Median Neuritis

**DOI:** 10.7759/cureus.57549

**Published:** 2024-04-03

**Authors:** Alexis M Ruppel, Mitchell J Lomis, Scarlett E Schneider

**Affiliations:** 1 Medicine, Augusta University Medical College of Georgia, Athens, USA; 2 Sports Medicine, Augusta University Medical College of Georgia, Athens, USA

**Keywords:** peripheral nerve disorders, orthopedic hand surgery, acute carpal tunnel syndrome, carpal tunnel syndome, median nerve injury

## Abstract

Acute median nerve neuropathy is most often due to trauma; acute carpal tunnel syndrome is considered a surgical emergency and must be ruled out. A right-hand dominant male presented to the emergency department with progressive unilateral pain and numbness in the median nerve distribution after experiencing a pop while doing pushups. The evaluation was limited to pain, but there was no gross deformity, and the distal right upper extremity was neurovascularly intact. All imaging was unremarkable. The patient received adequate pain control and complete resolution of symptoms. Despite presenting with symptoms congruent with possible carpal tunnel syndrome, the patient’s physical exam and imaging findings were inconsistent with the diagnosis. Acute median nerve neuritis is less commonly described, and no cases have been reported secondary to push-ups, but it should be considered in nontraumatic patients. With conservative management, patients can have complete resolution and no reoccurrence of symptoms.

## Introduction

Median nerve injuries are the result of acute traumatic, chronic micro traumatic, and/or compressive lesions [1]. The ultimate effect and deficits of trauma on the median nerve depend on the injury site, which may involve the palm, forearm, arm, or axilla. Injury of the median nerve can lead to motor, sensory, and vasomotor loss, with the majority of median nerve injuries occurring at the wrist [1]. Most median nerve injuries occur at the level of the carpal tunnel, which results in motor and sensory symptoms involving the thumb, index, middle, and radial aspect of the ring finger on the volar aspect of the hand [1]. When damage occurs at the level of or distal to the wrist, there is a sparing of symptoms of the thenar eminence because its innervation by the palmar cutaneous branch does not travel within the tunnel [1]. Acute carpal tunnel syndrome is a surgical emergency requiring urgent decompression [2,3]. Progressively worsening pain with the consistent sensory disturbances in the distribution described above are findings that will distinguish an acute carpal tunnel syndrome from less severe median nerve neurapraxia [2,4].

## Case presentation

A 57-year-old right-hand dominant male who works as a self-defense instructor presented to the emergency department (ED) with one day of severe right wrist pain and numbness. Early that morning, he was completing an intense workout during which he felt a “pop” while doing push-ups. Subsequently, he developed severe right wrist pain and numbness to his thumb, pointer, middle, and ring fingers. He initially presented to the orthopedic surgeon's office, who diagnosed him with a wrist sprain and placed him in a right wrist splint. His wrist pain and numbness continued progressing, prompting a revisit to the emergency department.

On examination in the ED, the patient's blood pressure was elevated at 174/86, with heart rate, temperature, and oxygen saturation within normal limits. His initial labs were unremarkable except for low sodium of 131 and elevated C-reactive protein (CRP) of 1.2.

The physical exam showed no gross forearm, wrist, hand, or finger deformity. The patient was tender to palpation of the volar distal forearm and wrist. Range of motion was limited to pain past 10 degrees of wrist flexion or extension. The patient noted wrist and distal forearm pain with terminal flexion and extension of the fingers. Range of motion, strength (EPL, FPL, FDS, FDP), sensation to light touch, and 2-point discrimination on the ulnar and radial aspects of the digits were all within normal limits. Capillary refill was < 2 seconds in all numbers.

**Figure 1:**
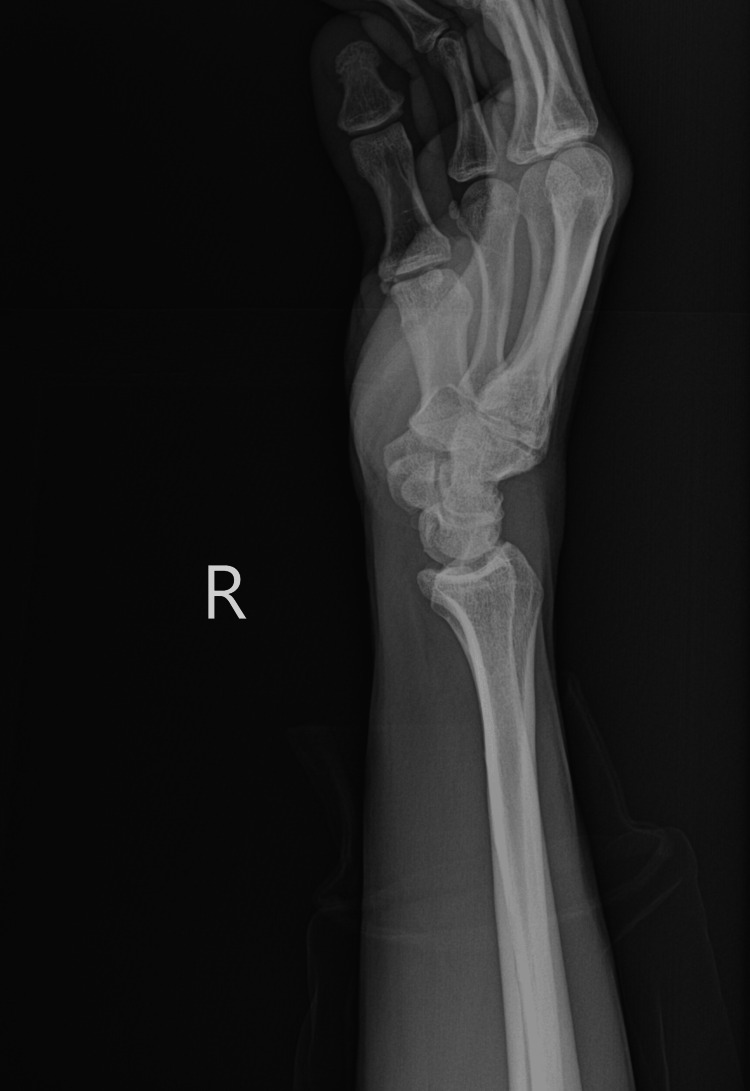
Lateral View Right Wrist X-ray

**Figure 2:**
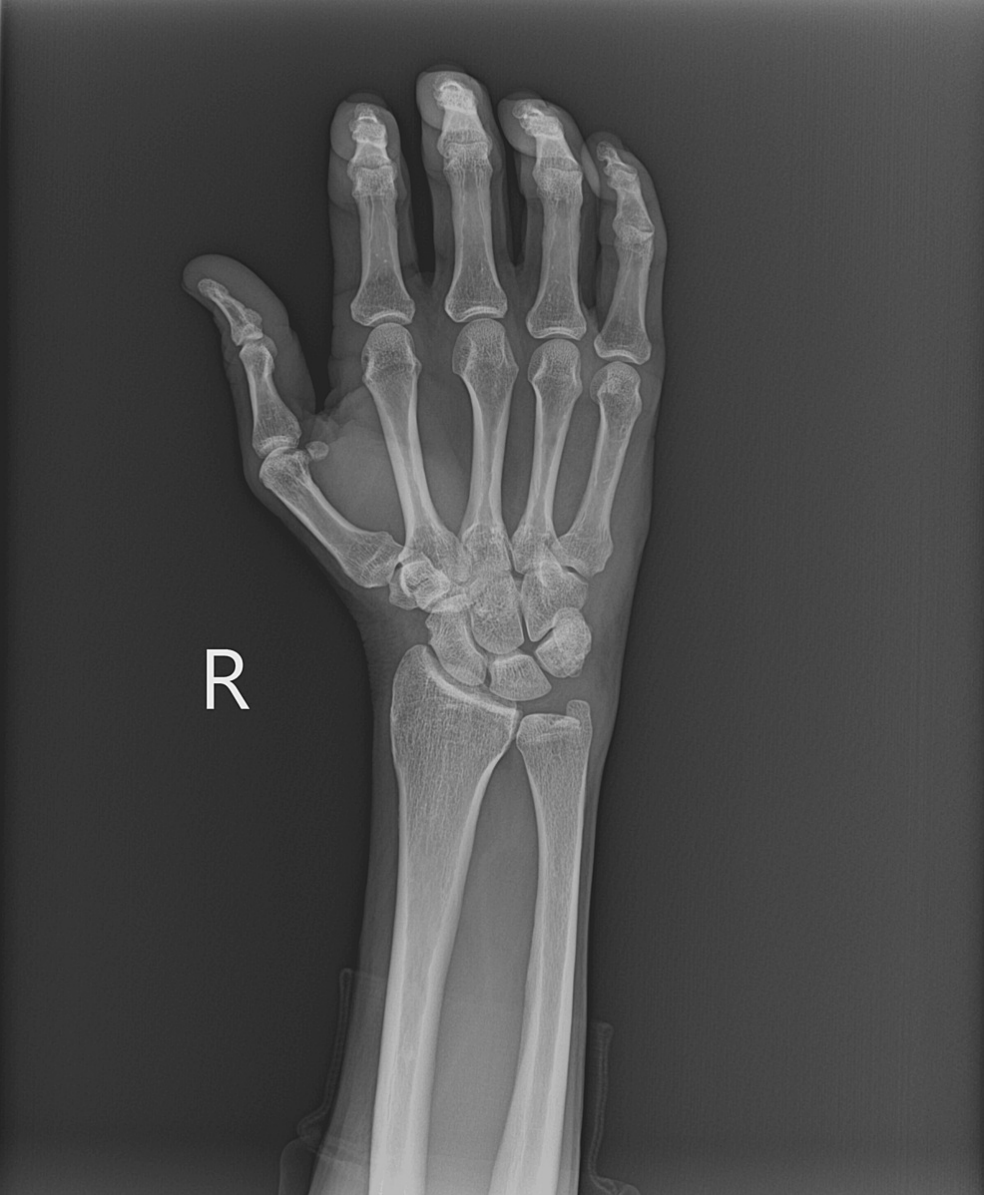
Posteroanterior View Right Wrist X-ray

**Figure 3:**
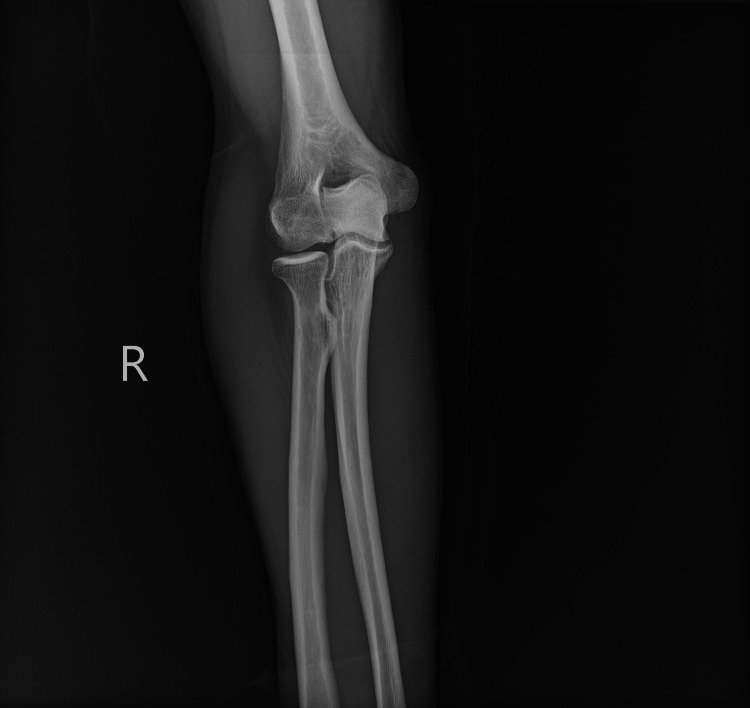
Posteroanterior View Right Forearm with Elbow Joint X-ray

**Figure 4:**
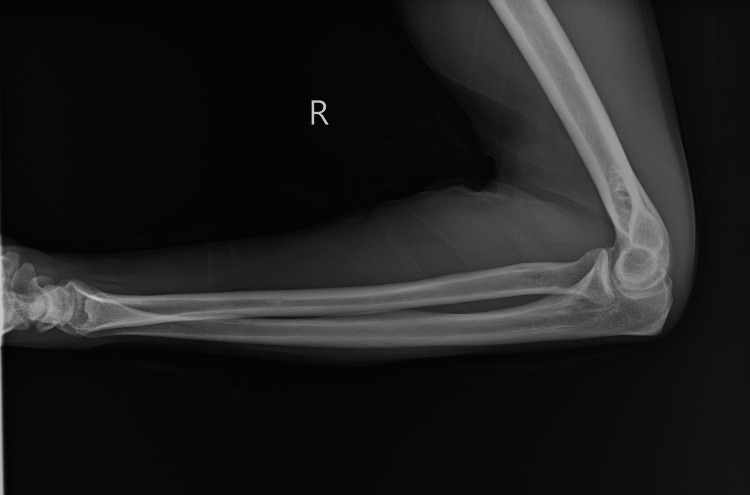
Lateral View Right Forearm with Wrist and Elbow Joints X-ray

Venous duplex ultrasound of the right upper extremity was negative for acute deep vein thrombosis. Multi-view X-rays of the right wrist and forearm (Figures 1-4) showed no fracture or dislocation; the radiocarpal, carpometacarpal, and intercarpal joints were normal in configuration and alignment. No osseous abnormalities were identified, and the periarticular soft tissues appeared normal. Computed tomography angiography of the right upper extremity with and without contrast showed no evidence of arterial dissection, hemodynamically significant stenosis, or occlusion.

During a subsequent physical exam, the patient was found to be in atrial flutter, presumptively due to pain, prompting admission to the hospital, and was started on metoprolol for rate control. The patient received Percocet 10mg-325mg scheduled every 6 hours and Tylenol 650 mg scheduled every 4 hours for pain. The next day vitals showed an improved blood pressure of 113/65.

A repeat physical examination the following day showed decreased pain with wrist flexion and extension. The patient was placed in a splint and discharged with gabapentin 300 mg twice daily and Prednisone 20 mg daily for neuropathic pain and inflammation, with recommendations for a one-week follow-up with orthopedics. The patient's pain and numbness progressively subsided over several days, with complete resolution by day three. Upon returning to work and exercise, the patient has not experienced any recurrent symptoms. Serial follow-ups at six months and one year were recommended to assess for potential long-term changes of the median nerve.

## Discussion

In the case presented, we propose the mechanism of injury is acute median neuritis secondary to chronic microtrauma from repetitive stretching and compression during self-defense maneuvers. This was then augmented and further provoked similarly while doing push-ups. Patients presenting with acute carpal tunnel syndrome typically offer a loss of two-point discrimination and increased compartment pressures [2]. These patients often also present with motor deficits in the median nerve distribution, including weakened thumb, pointer, middle, and ring finger flexion. After a delayed presentation to the emergency department, this patient's physical exam was inconsistent with acute carpal tunnel syndrome; however, the significant pain and numbness in a median nerve distribution point to an acute median nerve injury process. This patient's pain was severe enough to induce new-onset atrial flutter, which is rarely described in the setting of musculoskeletal injury without cardiac trauma. In an acute setting, this patient's pain level was more consistent with acute carpal tunnel or compartment syndrome. It is important for clinicians to know an injury to the median nerve, not secondary to traumatic compression neuropathy or increasing compartment pressures within the forearm, can be this severe in an otherwise healthy individual.

Because acute carpal tunnel syndrome is a surgical emergency, it must be distinguished from nerve contusion as a cause of acute post-traumatic median neuropathy [4]. Prompt diagnosis and treatment of acute injury to the median nerve after wrist trauma are paramount to a successful outcome [2]. Neuropathy, secondary to nerve contusion without coexisting acute carpal tunnel syndrome, can be treated initially by observation and pain control. Neuropathy occurs acutely at the time of injury because of trauma, secondary to fracture, or prolonged immobilization [2]. Although a median nerve pathology can be apparent via a strong history, there are several modalities that can aid in diagnosis [1]. Plain film images, with the use of a carpal tunnel view, can assist in diagnosis; ultrasound is another imaging modality increasingly used to diagnose nerve pathology [1]. Electromyography can also be utilized to monitor continued median nerve involvement. Patients similar to this one should undergo additional studies in the outpatient setting, including magnetic resonance imaging (MRI) and electromyography (EMG), to further investigate for evidence of chronic microtrauma or stretch injury to the median nerve. In the case of a patient with benign imaging and studies, the authors recommend treating patients with median neuritis with physical therapy, activity modification, and avoidance of provoking movements, such as the end range of motion wrist extension that is induced with pushups. There is a predominance of case reports describing acute carpal tunnel syndrome and acute median neuritis, and therefore further prospective studies should be directed towards better investigating recovery outcomes in both, which are seldom described and not well understood based on current data [5].

## Conclusions

Median nerve neuropraxia is a contusive stretch injury that occurs due to several etiologies. Often, due to trauma, patients demonstrate nonprogressive symptoms, but it is critical to rule out acute carpal tunnel syndrome before conservative management with expectant observation occurs. This case represents a clinically suggestive case of acute carpal tunnel but was determined to be less severe with the use of imaging. It is essential to understand that not all cases of acute carpal tunnel and neuropraxia are created equal despite often presenting similarly, and one must look at the overall picture rather than just one aspect of the clinical workup to provide the most appropriate care.

## References

[1] Dydyk AM, Negrete G, Sarwan G, Cascella M (2023). Median nerve injury. In: StatPearls [Internet].

[2] Gillig JD, White SD, Rachel JN (2016). Acute carpal tunnel syndrome: a review of current literature. Orthop Clin North Am.

[3] Holbrook HS, Hillesheim RA, Weller WJ (2022). Acute carpal tunnel syndrome and median nerve neurapraxia: a review. Orthop Clin North Am.

[4] Mack GR, McPherson SA, Lutz RB (1994). Acute median neuropathy after wrist trauma. The role of emergent carpal tunnel release. Clin Orthop Relat Res.

[5] Ku YC, Gannon M, Fang W, Norcini RC, Woodberry KM (2023). Management of acute carpal tunnel syndrome: a systematic review. J Hand Surg Glob Online.

